# Discovery and Analytical Validation of a Vocal Biomarker to Monitor Anosmia and Ageusia in Patients With COVID-19: Cross-sectional Study

**DOI:** 10.2196/35622

**Published:** 2022-11-08

**Authors:** Eduardo Higa, Abir Elbéji, Lu Zhang, Aurélie Fischer, Gloria A Aguayo, Petr V Nazarov, Guy Fagherazzi

**Affiliations:** 1 Deep Digital Phenotyping Research Unit Department of Population Health Luxembourg Institute of Health Strassen Luxembourg; 2 Bioinformatics Platform Quantitative Biology Unit Luxembourg Institute of Health Strassen Luxembourg

**Keywords:** vocal biomarker, COVID-19, ageusia, anosmia, loss of smell, loss of taste, digital assessment tool, digital health, medical informatics, telehealth, telemonitoring, biomarker, pandemic, symptoms, tool, disease, noninvasive, AI, artificial intelligence, digital, device

## Abstract

**Background:**

The COVID-19 disease has multiple symptoms, with anosmia and ageusia being the most prevalent, varying from 75% to 95% and from 50% to 80% of infected patients, respectively. An automatic assessment tool for these symptoms will help monitor the disease in a fast and noninvasive manner.

**Objective:**

We hypothesized that people with COVID-19 experiencing anosmia and ageusia had different voice features than those without such symptoms. Our objective was to develop an artificial intelligence pipeline to identify and internally validate a vocal biomarker of these symptoms for remotely monitoring them.

**Methods:**

This study used population-based data. Participants were assessed daily through a web-based questionnaire and asked to register 2 different types of voice recordings. They were adults (aged >18 years) who were confirmed by a polymerase chain reaction test to be positive for COVID-19 in Luxembourg and met the inclusion criteria. Statistical methods such as recursive feature elimination for dimensionality reduction, multiple statistical learning methods, and hypothesis tests were used throughout this study. The TRIPOD (Transparent Reporting of a multivariable prediction model for Individual Prognosis Or Diagnosis) Prediction Model Development checklist was used to structure the research.

**Results:**

This study included 259 participants. Younger (aged <35 years) and female participants showed higher rates of ageusia and anosmia. Participants were aged 41 (SD 13) years on average, and the data set was balanced for sex (female: 134/259, 51.7%; male: 125/259, 48.3%). The analyzed symptom was present in 94 (36.3%) out of 259 participants and in 450 (27.5%) out of 1636 audio recordings. In all, 2 machine learning models were built, one for Android and one for iOS devices, and both had high accuracy—88% for Android and 85% for iOS. The final biomarker was then calculated using these models and internally validated.

**Conclusions:**

This study demonstrates that people with COVID-19 who have anosmia and ageusia have different voice features from those without these symptoms. Upon further validation, these vocal biomarkers could be nested in digital devices to improve symptom assessment in clinical practice and enhance the telemonitoring of COVID-19–related symptoms.

**Trial Registration:**

Clinicaltrials.gov NCT04380987; https://clinicaltrials.gov/ct2/show/NCT04380987

## Introduction

In the context of the COVID-19 pandemic, declared by the World Health Organization in early March 2020, the fast and easy diagnosis of the disease has become an important concern. Anosmia, an olfactory dysfunction that leads to a temporary or permanent loss of olfaction, is present in 75% to 95% [1-3] of infected patients, whereas ageusia, a gustatory dysfunction resulting from the loss of functions of the tongue, is present in 50% to 80% [1,2,4,5] of infected people and can predict infection [6], depending on the virus strain and population characteristics. Proportionally, younger and female patients showed higher rates of these symptoms—a proven correlation due to differences in cytokine storms [5,7].

Monitoring these symptoms is highly needed and could be facilitated with an easy-to-use digital health solution. In individual who are infected but not tested, checking such symptoms could also serve as a rapid screening solution and suggest the realization of a test to limit the spread of the virus. There are also many concerns about the so-called Long COVID, where anosmia and ageusia are frequently reported [8]. A fast, noninvasive symptom assessment tool would be useful to better understand the whole spectrum of the disease and monitor Long COVID's evolution over time. Furthermore, these symptoms are associated with neurodegenerative diseases such as Parkinson and Alzheimer diseases [9,10] and can lead to multiple impacts, such as nutritional deficits [11].

The human voice is a rich medium that serves as a primary source of communication between individuals. Furthermore, talking is a uniquely human ability; it is one of the most natural and energy-efficient ways of interacting with each other. Slight alterations, for instance, due to a COVID-19–related symptom, are made by changes either in respiration, phonation, or articulation—the 3-stage process of voice production [12]—which will result in variations of pitch, tone, fundamental frequency, and many other aspects of our voice. Recent developments in audio signal processing and artificial intelligence methods have enabled a more refined and in-depth voice features analysis that surpasses the human level of perception and can solve complex problems in the health care domain.

This study aimed to test the hypothesis that anosmia and ageusia following a SARS-CoV-2 infection can result in modifications in voice production that could help detect and monitor these specific symptoms. To achieve our objective, we used data from the prospective Predi-COVID cohort study, where both voice and COVID-19–related symptoms were frequently recorded. We analyzed voice signals, built panels of vocal biomarkers, and internally validated them using the developed prediction models.

## Methods

### Study Population

This study used data from the Predi-COVID cohort [13]—a prospective, hybrid cohort started in May 2020 composed of adult patients (aged >18 years) who were confirmed, by a polymerase chain reaction test, to be positive for COVID-19 in Luxemburg, both in and out of the hospital.

The first contact with potential participants was made via phone by collaborators from the Health Inspection. Those who agreed to take part were contacted by an experienced nurse or clinical research associate from the Clinical and Epidemiological Investigation Center, who explained the study and organized visits at home or the hospital, and informed consent for participation was obtained.

Through the first 14 days following inclusion, participants were assessed daily through a web-based questionnaire. A subcohort agreed to be digitally followed by a digital app that was dedicated to voice recording in cohort studies. To guarantee a minimum quality standard, participants were instructed to register the audio in a calm place while keeping a specific distance from the microphone**.** An audio example of what was expected was also available.

Each day, 2 types of voice recordings were performed. In the first recording, called Type 1 audio, participants had to read an extract from the Declaration of Human Rights, Article 25, paragraph 1 (Multimedia Appendix 1) in their preferred language: French, German, English, or Portuguese; and in the second recording, called Type 2 audio, they were asked to hold the “[a]” vowel phonation without breathing as long as they could. For this analysis, we considered only voice recordings from the first 2 weeks after inclusion where the symptoms were collected regularly. Since the study is in a real-life setting, the number of vocal samples per participant may have differed.

### Ethics Approval

The study was approved by the National Research Ethics Committee of Luxembourg (study 202003/07) in April 2020 and is registered on ClinicalTrials.gov (NCT04380987).

### Inclusion Criteria

All participants who had no missing data on sex, information on the studied outcome, and both types of audio recordings on the same day during the first 14 days of follow-up were included in the model.

### Anosmia and Ageusia

In this study, both anosmia and ageusia were the outcomes and were united in a single variable based on the participant’s perception. The specific question was the following: “Did you notice a strong decrease or a loss of taste or smell?” The possible answers were “yes” or “no.” Since the loss of smell can substantially affect taste functions [14], uniting the 2 symptoms is expected to be a more realistic strategy because the outcome is self-reported, and it would not be easy for the participant to clearly distinguish between ageusia and anosmia.

### Prediction Data

The prediction models were based on both Type 1 and Type 2 voice recordings to predict the outcome. To maximize the information given to the model, both types were concatenated and used as a single input to the learning model. The audio format and recording settings varied depending on the operating system of the smartphone used to record it: Android devices were registered in 3gp format, whereas iOS devices were registered in m4a format. These 2 formats were also analyzed separately to create predictive models for each type of operating system.

### Voice Signal Treatment

**Figure 1:**
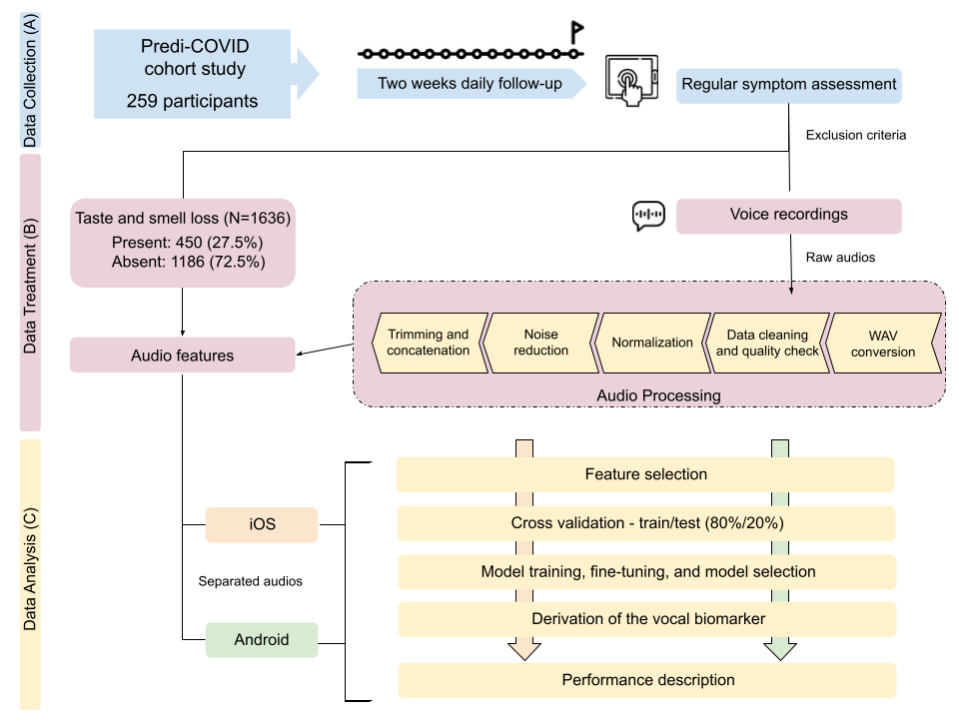
Learning pipeline to the discovery of biomarkers. (A) Data collection from Predi-COVID and exclusion criteria. (B) Data treatment of audio data and studied outcome. (C) Data analysis for both audio formats done in parallel.

The audios were preprocessed to remove poorly recorded or corrupted files, and the remaining ones were then normalized and cleaned for noise. Type 1 and Type 2 audios were both sampled with an 8000 Hz sample rate, as different rates did not significantly improve the model. Audios were then concatenated, which resulted in a final sample from which the features were extracted. The pipeline can be found in Figure 1.

### OpenSMILE

The Munich Open-Source Media Interpretation by Large Feature-Space Extraction (openSMILE) is a modular and flexible research-only toolkit for extracting features for signal processing and machine learning applications. It is widely used in the speech recognition community, the area of affective computing, and music information retrieval [15]. The package provides many functionalities, such as windowing functions, resampling, and fast Fourier transform. It can extract a wide range of features including frame energy, Mel-frequency cepstral coefficients, loudness, jitter, shimmer, and many others. The specific openSMILE feature set is the same as that used in The Interspeech 2016 Computational Paralinguistics Challenge [16], originally chosen to assess sentiments through the voice. Within it, there are 2 feature levels: functionals, which gather much more detailed information and reach up to 6473 different features; and low-level descriptors, measures that are closely related to the signal and reach up to 66 features [17]. The latter feature level is embedded in the functional features, and the full set of feature categories is shown in Multimedia Appendix 2.

### Recursive Feature Elimination

Recursive feature elimination (RFE) is a dimensionality reduction method that recursively ranks features according to a measure of importance defined by another classifier (linear regression and random forests, for example), and at each iteration, the ones with the lowest rank are removed until the desired number is reached [18]. The minimum number of features was set to 10, a linear regression was used to define the weights, and 25 features were removed at each iteration (step=25). This process was performed using 10-fold cross-validation.

### Statistical Analysis Methods

Chi-square test and Student *t* test (2-tailed) were used in this study. We applied standard machine learning algorithms that work with structured data to analyze the extracted features. Random forests [19], k-nearest neighbors (KNN) [20], and support vector machines [21] were used to avoid biases from a single predictor and test different approaches on the same data.

All hyperparameters were hyper tuned using grid search from *scikit-learn* (version 0.22.2) [22], maximizing the weighted area under the receiver operating characteristic curve (ROC AUC). The data were divided into a 60%/20%/20% proportion for training, validation, and testing, respectively. To evaluate its sensibility, 10-fold cross-validation was first performed on the training set to analyze the dispersion of the metrics, and then the final model was built on the testing set.

The final model was chosen based on the following metrics: precision, recall, *F*-measure, and accuracy. Given the nature of the problem, we assumed that having false negatives was worse than having false positives, since one can develop severe symptoms and continue to spread the virus if misclassified, so the recall for those positive to the studied outcome should be maximized. The weighted ROC AUC was also taken into account since it indicates the overall performance of the model in terms of its accuracy at various diagnostic thresholds used to discriminate between 2 classes [23].

To derive the vocal biomarker from the prediction model, we used the final probability of being classified as having anosmia or ageusia; its distribution was further evaluated in both groups.

## Results

### Descriptive Data

After excluding all data that did not meet the inclusion criteria, we used descriptive statistics to characterize the study participants. The final study population had a total of 259 participants, and age, sex, and BMI were associated with the outcome (*P*<.001, *P*<.001, *P*<.001, respectively). Younger (aged <35 years) and female participants showed higher rates of ageusia and anosmia.

Participants were aged 41 (SD 13) years on average with a BMI of 25.4 (SD 4.6)—the intersection between normal weight and overweight [24]. Antibiotics intake, asthma, and smoking were highly unbalanced clinical features (present in n=29, 11.2%; n=10, 3.9%; and n=177, 68.3% of participants, respectively). The data set was balanced for sex (female: n=134, 51.7%; male: n=125, 48.3%), and the analyzed symptom was present in 94 (36.3%) out of 259 participants and in 450 (27.5%) out of 1636 of audio recordings. This result occurs due to a variation in the number of recordings per participant, with each one having an average of 6 audio recordings. Finally, Type 1 audio had an average length of 28.5 s, whereas Type 2 audio had an average length of 18.9 s.

**Figure 2:**
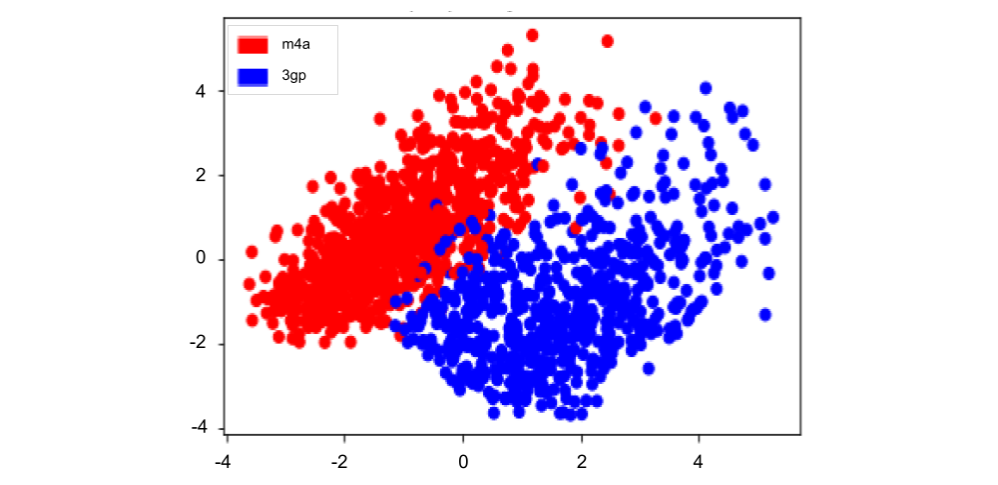
Sample plot with linear separation between 3gp and m4a audio formats. Principal component analysis was used on the extracted features, and the first 2 dimensions were used to plot the samples.

As the audio format was linearly separable when analyzing the outcome, shown in Figure 2, they were separated in the analysis. When divided by audio format, no significant difference was found between the 2 sets of participants. Clinical features and audio data can be seen in Tables 1-2.

**Table 1:** Description of the participants, containing clinical data to characterize the general population of the study and the loss of smell and taste. All categorical data are represented as the total number and its percentage.

Description		Total (N=259)		Audio format, operating system			*P* value^a^	
				m4a, iOS (n=161)	3gp, Android (n=98)			
**Symptom, n (%)**								.51
	Normal taste and smell		165 (63.7)	105 (65.2)	60 (61.2)			
	Loss of taste and smell		94 (36.3)	56 (34.8)	38 (38.8)			
**Sex, n (%)**								.14
	Female		134 (51.7)	89 (55.3)	45 (45.9)			
	Male		125 (48.3)	72 (44.7)	53 (54.1)			
**Antibiotic, n (%)**								.42
	No		230 (88.8)	141 (87.6)	89 (90.8)			
	Yes		29 (11.2)	20 (12.4)	9 (9.2)			
**Asthma, n (%)**								.88
	No		249 (96.1)	155 (96.3)	94 (95.9)			
	Yes		10 (3.9)	6 (3.7)	4 (4.1)			
**Smoking, n (%)**								.85
	Yes		177 (68.3)	112 (69.6)	65 (66.3)			
	Never		44 (17)	26 (16.1)	18 (18.4)			
	Former smoker		38 (14.7)	23 (14.3)	15 (15.3)			
Age (years), mean (SD)		40.6 (12.7)		40.6 (13.4)	40.7 (11.5)	.93		
BMI (kg/m²), mean (SD)		25.4 (4.6)		25.4 (4.9)	25.5 (4.1)	.80		

^a^All *P* values were calculated through chi-square or Student *t* test between m4a and 3gp formats.

**Table 2:** Description of the audio samples, with their general information.

Description		Total (N=1636)		Audio format, operating system			*P* value^a^	
				m4a, iOS (n=999)	3gp, Android (n=637)			
**Audio samples per symptom, n (%)**								.06
	Normal taste and smell		1186 (72.5)	741 (74.2)	445 (69.9)			
	Loss of taste and smell		450 (27.5)	258 (25.8)	192 (30.1)			
Number of audio samples per participant, mean (SD)		6.3 (4.5)		6.2 (4.4)	6.5 (4.6)	—^b^		
Text reading duration (s), mean (SD)		28.5 (4.1)		28.3 (4.1)	28.9 (4.2)	—		
Vowel phonation duration (s), mean (SD)		18.9 (6.8)		18.2 (6.6)	20 (7.1)	—		

^a^All *P* values were calculated through chi-square or Student *t* test between m4a and 3gp formats.

^b^Not available.

### Feature Extraction

We extracted 6473 features from the concatenated audios. Constant features throughout all the audios were removed from the analysis (50 for Android and 49 for iOS). A RFE method was used to find the best number of features (Multimedia Appendix 3). For 3gp and m4a audios, we selected 3248 and 849 features, respectively.

After extraction, a density plot for the low-level descriptors was made, as shown in Multimedia Appendices 4-5. It can be seen that the distribution of the variables varies depending on the outcome, which reinforces the hypothesis that there are vocal changes related to COVID-19 infection.

### Prediction Models’ Performances

The algorithms were first hyper tuned and then trained on all the extracted features and the ones selected through *RFECV*. All models used an 80%/20% stratified proportion for training and testing, respectively, and 10-fold cross-validation was used to assess its sensitivity. The *numpy* seed and the random state of all processes were set to 42 to assure reproducibility, and the samples were weighted to correct the models for unbalanced data.

Models trained on all features had an overall lower performance than those trained with selected features, mainly due to the removal of noise and correlated features (complementary information). The final models for the 3 tested learning algorithms are shown in Table 3. For both formats of audio, we identified KNN as the best method—showing better performances. The AUC was used to choose the best algorithm, and in the end, 3gp had an AUC of 87%, whereas m4a had an AUC of 80%. The specific hyperparameters for each algorithm can be found in Multimedia Appendix 6.

The final models for classifying the loss of taste and smell were KNN for both audio formats and presented a good weighted precision (88% for Android and 85% for iOS), weighted recall (88% for Android and 85% for iOS), and weighted AUC (87% for Android and 80% for iOS). The main difference between the 2 final models is on the recall for the symptomatic class, which was to be maximized (82% for Android and 69% for iOS).

**Figure 3:**
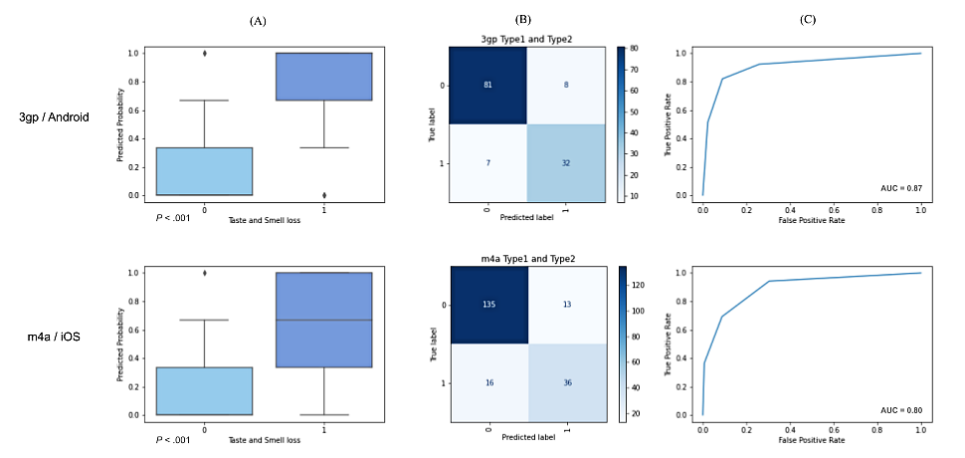
Final models for each audio format. (A) Biomarkers and *P* values from two-sided student's t-test for the presence of anosmia and ageusia were calculated using the probability of classifying as positive. (B) Confusion matrix of the best model. (c) ROC AUC curve. Class 0 represents absence of symptoms and Class 1 the presence of it. ROC AUC: area under the receiver operating characteristic curve.

The final vocal biomarker of loss of taste consisted of the probability of being classified as having the symptoms, calculated from the combination of all features selected for each audio format. Its range is shown in Figure 3A, and there was a significant difference between the distribution of probabilities for both 3gp and m4a formats (*P*<.001 and *P*<.001 respectively), which confirms that the model can statistically distinguish the 2 possible conditions, as the probability distribution differs between outcomes.

Figure 3 also presents the confusion matrix for the best classifiers, which shows that they are slightly better in correctly classifying the absence of symptoms than its presence. Additionally, the ROC AUC for each best model is plotted, proving its good learning thresholds.

**Table 3:** Performance for the 3 different learning methods for each audio format^a^.

Audio format (number of selected features), algorithm			Weighted precision		Weighted recall		Recall 1		Accuracy	Weighted AUC^b^	10-fold AUC (SD)
**3gp (n=3248)**											
	*KNN^c^*	*0.88*		*0.88*		*0.82*		*0.88*		*0.87*	*0.89 (0.05)*
	Random forest	0.77		0.77		0.33		0.77		0.64	0.86 (0.03)
	SVM^d^	0.81		0.81		0.64		0.81		0.76	0.87 (0.03)
**m4a (n=849)**											
	*KNN*	*0.85*		*0.85*		*0.69*		*0.85*		*0.80*	*0.89 (0.01)*
	Random Forest	0.75		0.77		0.30		0.78		0.70	0.76 (0.02)
	SVM	0.78		0.79		0.52		0.79		0.70	0.90 (0.01)

^a^The final model was selected using weighted AUC and is highlighted in italics. Cross-validation was used in the training set as a validation method, and the final model on the testing set showed good adherence to it. The other differences in k-fold and weighted AUC are due to differences in the testing and training set sizes.

^b^AUC: area under the curve.

^c^KNN: k-nearest neighbors.

^d^SVM: support vector machines.

## Discussion

### Principal Findings

In this study, we trained artificial intelligence–based algorithms to predict the presence of ageusia and anosmia in patients with COVID-19. In total, 2 predictive models were created based on each smartphone operating system (iOS or Android). We derived 2 sets of vocal biomarkers from these predictive models that should be used together as a single classifier. The biomarkers were then calculated and, after an external validation, can be used to accurately identify patients who present a loss of taste and smell.

### Biological Background

Voice is a proven source of medical information, can be easily recorded on a large scale through smart devices [25], and can be easily used to build personalized corpora [26]. Studies have shown great results in the early diagnosis of neurological disorders such as Parkinson disease [27,28], Alzheimer disease [29], and mild cognitive impairment [30,31], since they directly alter the voice, but also in nonneurological conditions such as cardiometabolic [32] and pulmonary [33] diseases. It is important to note that the analysis in this study is new since examples in the literature only analyze short audios (shorter than 5 s) and usually use coughs and other sources of sound [34-36].

Anosmia and ageusia are common COVID-19 symptoms that usually emerge after 5 days of infection [37]. The upper part of the respiratory tract, mainly the olfactory epithelium, is rich in ACE2 and TMPRSS2, 2 main SARS-CoV-2 receptors [38]. Olfactory sensory neurons, on the other hand, were not found to express these receptors, which indicates that the disease itself probably does not directly alter the mechanisms of smell and taste. The infection of support cells, mainly sustentacular and Bowman glands, of these regions and their subsequent malfunction result in alterations in the environment, causing local neuronal death and the final symptom of loss of taste and smell [38,39].

Given that there is no neuronal causality between the loss of taste and smell and voice production, the main pathway in the voice likely involves mechanical influences of COVID-19 infection. The disease alters various systems, such as the respiratory, cardiovascular, and gastrointestinal systems, that if impaired, can directly impact voice characteristics. In mild cases, general symptoms frequently associated with the loss of taste and smell such as dry coughs, insufficient airflow, and pulmonary status also directly affect the production of sounds, resulting in variations that can be used to predict the loss of taste and smell [12].

### Strengths and Limitations

The main strengths of this study come from the fact that all participants were confirmed to be positive for COVID-19 by a polymerase chain reaction test. Besides, the majority of the published studies relied on data from hospitalized patients. Therefore, having a cohort of participants mostly at home brings complementary information on the entire spectrum of the disease severity of COVID-19 (from asymptomatic to severe cases). The audio recording is based on a standardized text that has an official translation in many languages, which ensures the high reproducibility of the task in future studies in other countries. The second audio type is a sustained vowel and is, therefore, language-independent and allows analysis without risks of biases due to different articulatory factors, speaking rates, stress, intonations, or any other characteristics that may vary between languages.

This study also has limitations. The recordings are performed in a real-life, noncontrolled environment, which may increase the variability in the quality of the voice recordings. However, since the ultimate objective is to deploy a digital health solution, we cannot rely on well-controlled audio recordings based on a unique device to train the algorithms and should integrate from scratch the diversity of devices and audio recording environments. This study integrates a mixture of different languages in the cohort, but the developed vocal biomarkers cannot be applied to other languages yet. Even though the text is the same, different languages and accents might result in different model performances. Additional external validation studies in other populations that are not well represented in this study (young people) are required at this stage.

In conclusion, we demonstrated that people with COVID-19 who had anosmia and ageusia had different voice features and that it is feasible to accurately predict the presence or absence of this frequent COVID-19 symptom with just a few seconds of the individual’s voice. The derived vocal biomarker is strongly associated with the presence of the symptom and could soon be integrated into digital health solutions to help clinicians enhance their consultations or in telemonitoring solutions for remote monitoring. Further external validation studies in other populations and languages are now required.

## Appendix

Multimedia Appendix 1 Standardized, prespecified text to be read by study participants to collect voice recordings.

Multimedia Appendix 2 OpenSMILE categories of extracted features for the 2 feature levels. A detailed description can be found on the web [15].

Multimedia Appendix 3 Variation of the AUC performance when varying the number of selected features using RFECV. AUC: area under the curve.

Multimedia Appendix 4 Density plot of the low-level descriptors for 3gp audio format.

Multimedia Appendix 5 Density plot of the low-level descriptors for m4a audio format.

Multimedia Appendix 6 Hyperparameters for the best algorithms. The random state seed was always set to 42 and the maximum number of iterations to 10000. The implementation of scikit-learn (version 0.22.2) was used.

## Abbreviations

KNN: k-nearest neighbors
openSMILE: Open-Source Media Interpretation by Large Feature-Space Extraction
RFE: recursive feature elimination
ROC AUC: area under the receiver operating characteristic curve
