# Supplementary material for: Discovery and Analytical Validation of a Vocal Biomarker to Monitor Anosmia and Ageusia in Patients With COVID-19: Cross-sectional Study
Source: JMIR Med Inform. 2022 Nov 8;10(11):e35622. doi: 10.2196/35622 (PMC9645416; doi:10.2196/35622)
Supplement: Multimedia Appendix 2 [file medinform_v10i11e35622_app2.pdf]

OpenSMILE categories of extracted features for the two feature levels. A detailed description can be found at https://audeering.github.io/opensmile/about.html.

Low level descriptors	Functional
Frame Energy	Extreme values and positions
Frame Intensity / Loudness (approximation)	Means (arithmetic, quadratic, geometric)
Critical Band spectra (Mel/Bark/Octave, triangular masking filters)	Moments (standard deviation, variance, kurtosis, skewness)
Mel-/Bark-Frequency-Cepstral Coefficients (MFCC)	Percentiles and percentile ranges
Auditory Spectra	Regression (linear and quadratic approximation, regression error)
Loudness approximated from auditory spectra	Centroid
Perceptual Linear Predictive (PLP) Coefficients	Peaks
Perceptual Linear Predictive Cepstral Coefficients (PLP-CC)	Segments
Linear Predictive Coefficients (LPC)	Sample values
Line Spectral Pairs (LSP, aka. LSF)	Times/durations
Fundamental Frequency (via ACF/Cepstrum method and via Subharmonic-Summation (SHS))	Onsets/Offsets
Probability of Voicing from ACF and SHS spectrum peak	Discrete Cosine Transformation (DCT)
Voice-Quality: Jitter and Shimmer	Zero Crossings
Formant frequencies and bandwidths	Linear Predictive Coding (LPC) coefficients and gain
Zero and Mean Crossing rate	
Spectral features (arbitrary band energies, roll-off points, centroid, entropy, maxpos, minpos, variance (= spread), skewness, kurtosis, slope)	
Psychoacoustic sharpness, spectral harmonicity	
CHROMA (octave-warped semitone spectra) and CENS features (energy-normalised and smoothed CHROMA)	
CHROMA-derived features for Chord and Key recognition	
F0 Harmonics ratios	
