# Supplementary figures and images for: Discovery and Analytical Validation of a Vocal Biomarker to Monitor Anosmia and Ageusia in Patients With COVID-19: Cross-sectional Study
Source: JMIR Med Inform. 2022 Nov 8;10(11):e35622. doi: 10.2196/35622 (PMC9645416; doi:10.2196/35622)

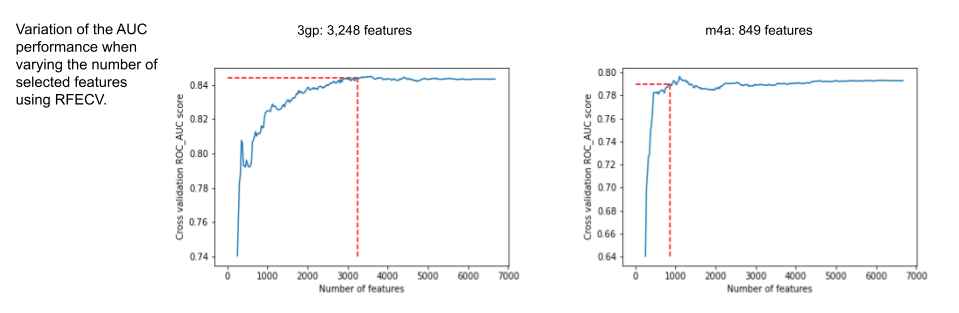

Supplement: Multimedia Appendix 3 [file medinform_v10i11e35622_app3.png]

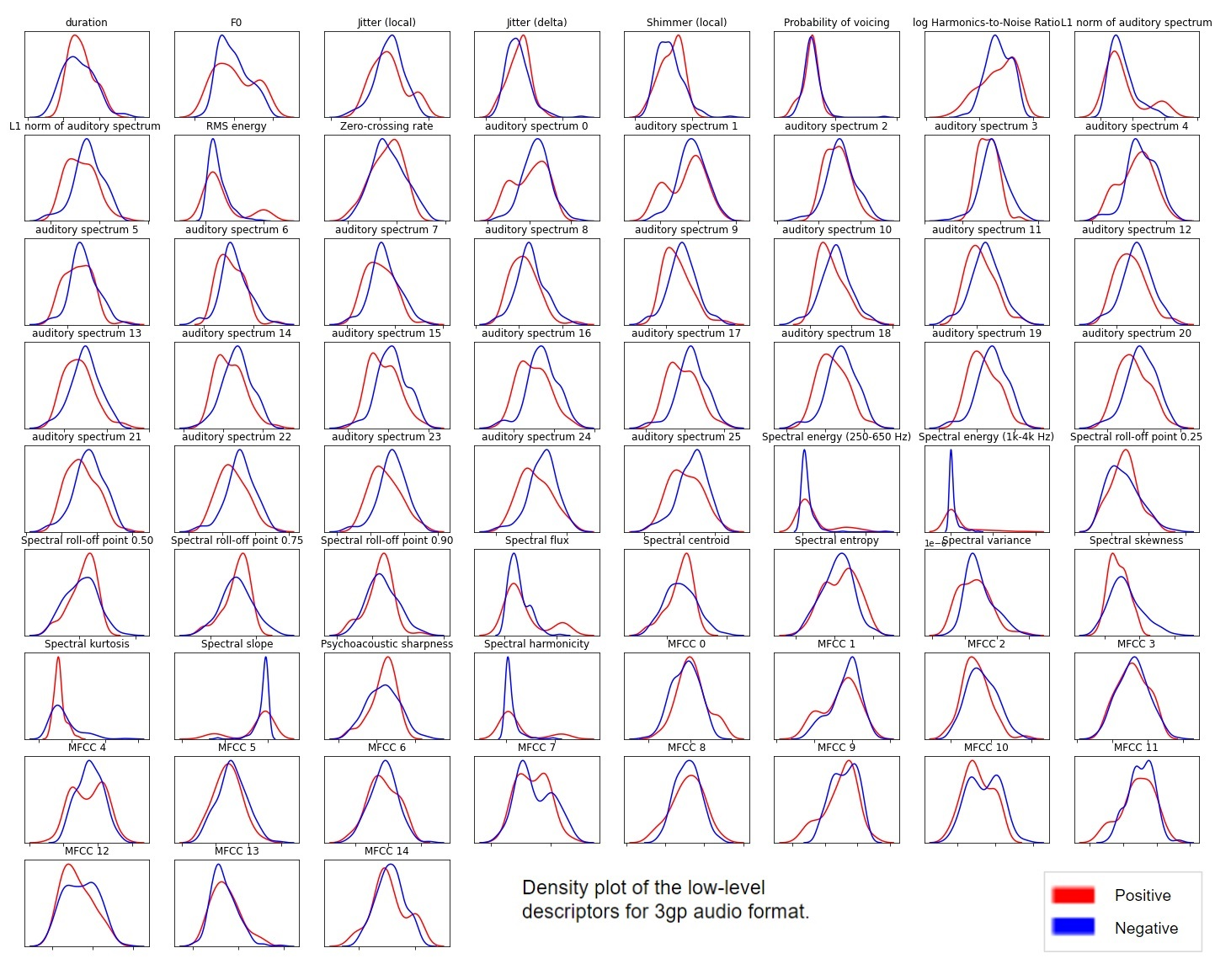

Supplement: Multimedia Appendix 4 [file medinform_v10i11e35622_app4.png]

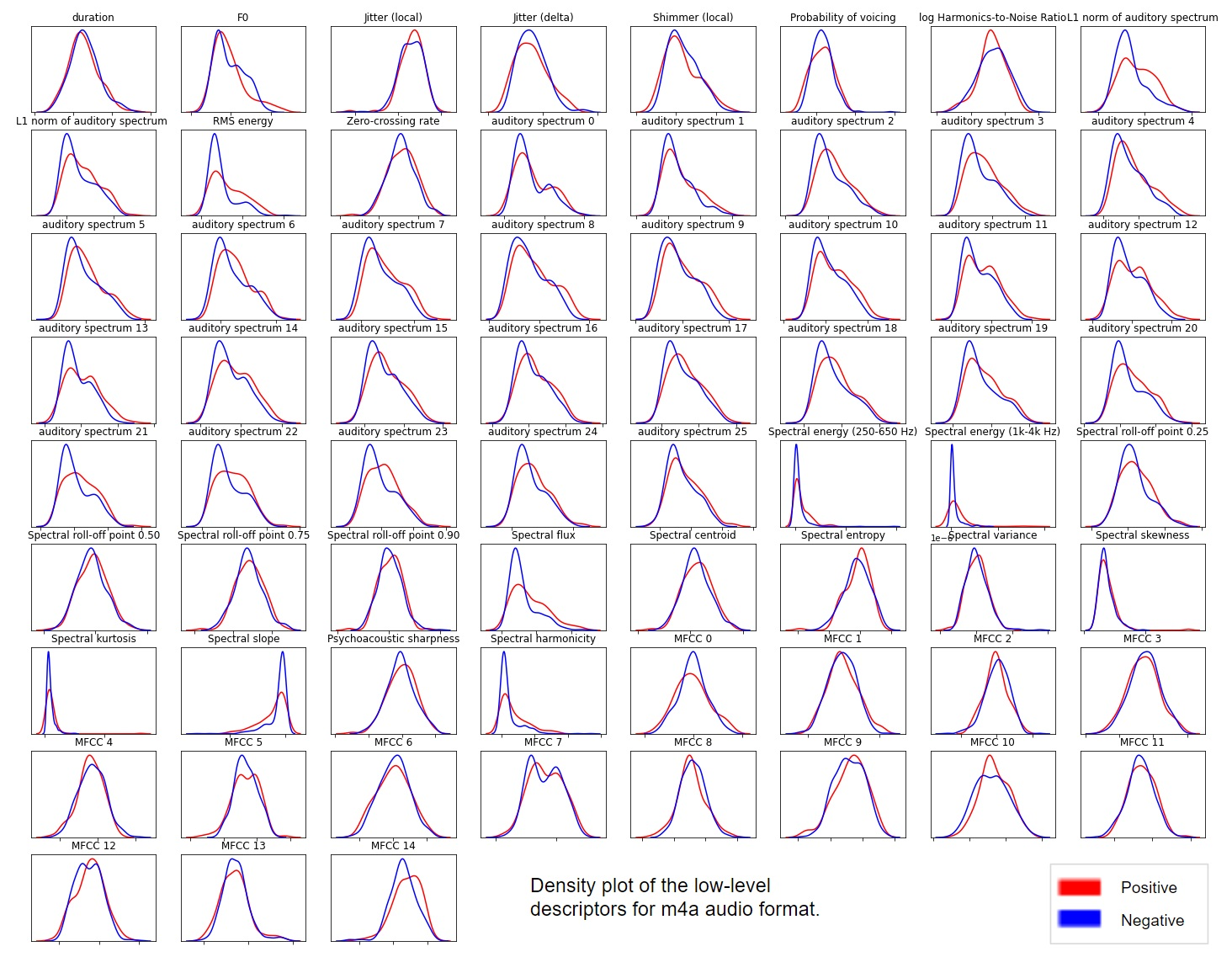

Supplement: Multimedia Appendix 5 [file medinform_v10i11e35622_app5.png]
