# Supplementary material for: Discovery and Analytical Validation of a Vocal Biomarker to Monitor Anosmia and Ageusia in Patients With COVID-19: Cross-sectional Study
Source: JMIR Med Inform. 2022 Nov 8;10(11):e35622. doi: 10.2196/35622 (PMC9645416; doi:10.2196/35622)
Supplement: Multimedia Appendix 6 [file medinform_v10i11e35622_app6.pdf]

Hyperparameters for the best algorithms. The random state seed was always set to 42 and the maximum number of iterations to 10000. The implementation of scikit-learn v0.22.2 was used.

Audio format	Number of features	Algorithm	Algorithm	Distance	Number of Neighbors	Weights
3gp	3248	KNN	auto	euclidean	3	uniform
m4a	849		auto	manhattan	3	uniform

Audio format	Number of features	Algorithm	Criterion	Max depth	Minimum sample split	n_estimators
3gp	3248	Random Forest	entropy	10	10	100
m4a	849		entropy	10	10	25

Audio format	Number of features	Algorithm	Kernel	C	Degree	Tolerance	Gamma
3gp	3248	SVM	linear	0.1	3	0.001	-
m4a	849		sigmoid	1000	3	0.001	auto
